# A Real-World Rheumatology Registry and Research Consortium: The German RheumaDatenRhePort (RHADAR) Registry

**DOI:** 10.2196/28164

**Published:** 2021-05-20

**Authors:** Stefan Kleinert, Peter Bartz-Bazzanella, Cay von der Decken, Johannes Knitza, Torsten Witte, Sándor P Fekete, Matthias Konitzny, Alexander Zink, Georg Gauler, Patrick Wurth, Peer Aries, Kirsten Karberg, Christoph Kuhn, Florian Schuch, Susanna Späthling-Mestekemper, Wolfgang Vorbrüggen, Matthias Englbrecht, Martin Welcker, RHADAR Group

**Affiliations:** 1 Praxisgemeinschaft Rheumatologie-Nephrologie Erlangen Germany; 2 Medizinische Klinik 3, Rheumatology/Immunology Universitätsklinik Würzburg Würzburg Germany; 3 Medizinisches Versorgungszentrum Stolberg Stolberg Germany; 4 Klinik für Internistische Rheumatologie Rhein-Maas-Klinikum Würselen Germany; 5 Department of Internal Medicine 3 – Rheumatology and Immunology Friedrich-Alexander-University Erlangen-Nürnberg University Hospital Erlangen Erlangen Germany; 6 Department of Rheumatology and Clinical Immunology Hanover Medical School Hanover Germany; 7 Department of Computer Science TU Braunschweig Braunschweig Germany; 8 Department of Dermatology and Allergy School of Medicine Technical University of Munich Munich Germany; 9 Rheumatologische Schwerpunktpraxis Osnabrück Germany; 10 Rheumatologie im Struenseehaus Hamburg Germany; 11 Praxis für Rheumatologie und Innere Medizin Berlin Germany; 12 Praxis für Rheumatologie Karlsruhe Germany; 13 Rheumatologist in Group Practice Munich Germany; 14 Verein zur Förderung der Rheumatologie eV Würselen Germany; 15 Freelance Healthcare Data Scientist Eckental Germany; 16 Medizinisches Versorgungszentrum für Rheumatologie Dr M Welcker GmbH Planegg Germany; 17 see Acknowledgments

**Keywords:** registry, rheumatology, real-world data, symptom checker, patient-reported outcomes

## Abstract

Real-world data are crucial to continuously improve the management of patients with rheumatic and musculoskeletal diseases (RMDs). The German RheumaDatenRhePort (RHADAR) registry encompasses a network of rheumatologists and researchers in Germany providing pseudonymized real-world patient data and allowing timely and continuous improvement in the care of RMD patients. The RHADAR modules allow automated anamnesis and adaptive coordination of appointments regarding individual urgency levels. Further modules focus on the collection and integration of electronic patient-reported outcomes in between consultations. The digital RHADAR modules ultimately allow a patient-centered adaptive approach to integrated medical care starting as early as possible in the disease course. Such a closed-loop system consisting of various modules along the whole patient pathway enables comprehensive and timely patient management in an unprecedented manner.

## Current Challenges in Rheumatology

The efficacy and safety of treatments for patients with rheumatic and musculoskeletal diseases (RMDs) have continuously improved during previous decades. However, the combination of a declining number of rheumatologists [1] on the one hand and a rising life expectancy in developed countries on the other imposes a significant challenge to rheumatology care nowadays. The growing need for a professional evaluation of common symptoms, such as joint pain [2,3], can hardly be met by the number of available rheumatologists. Besides the lack of the latter, multiple factors contribute to the omnipresent diagnostic delay in rheumatology. Unspecific rheumatic symptoms are challenging to patients [4], general practitioners [5], and experienced rheumatologists [6] alike. Even the most advanced and innovative therapies cannot reverse the health impairment that manifests due to diagnostic delay. “Time is joint” depicts the concept of a timely diagnosis as an indispensable goal in rheumatology. Focusing on this goal could be more cost-effective and generate better outcomes than the development of new RMD drugs.

The shortage of rheumatologists translates to reduced doctor-patient time, especially for follow-up appointments. On average, patients have appointments every 3 to 6 months, while rheumatologists spend 15 minutes for each consultation. In complex diseases like RMDs, the long-term treatment effectiveness cannot be deduced from a single laboratory result like HbA_1c_ in diabetes mellitus. The current monitoring approach in rheumatology relies mainly on the collection of laboratory, clinical, and anamnestic snapshots. This “black box” monitoring approach hardly represents modern personalized medicine. Apart from diagnostic delay, timely treatment adaptions are hampered by the status-quo approach.

Rheumatology encompasses various rare diseases, with limited evidence aiding clinicians in their treatment decisions. Despite the growing number of therapeutic options and treatment guidelines, patients frequently feel like guinea pigs, with prescribed anti-inflammatory treatments not showing the expected therapeutic effect. Thus, clinical evidence supported by real-world data is needed to improve personalized evidence-based treatment decisions.

## Potential of a Digital Patient Pathway in Rheumatology

The potential of digitalization in rheumatology has been depicted in recent publications [7-14]. The European League Against Rheumatism (EULAR) recommends that patients experiencing morning stiffness, joint pain, or swollen joints should see a rheumatologist no later than 6 weeks after symptom onset [15]. A symptom-based, patient-centered, digital screening approach could allow the successful implementation of this guideline. This low burden approach could effectively cut diagnostic delay. The omnipresence of smartphones in the RMD population [14] (irrespective of age) and instinctive digital symptom-based search for solutions [2,3,11,14] represent an immense potential to guide and triage patient streams effectively.

Electronic patient-reported outcomes (ePROs) allow for an individual, continuous, flexible, asynchronous, and need-adapted patient follow-up. Only few rheumatologists are currently using ePROs in clinical routine, mainly due to unawareness of suitable software solutions [13]. Similarly, only few RMD patients are aware of useful rheumatology-specific medical applications [14]. However, rheumatologists and patients are motivated to use ePROs and rheumatology-specific applications in the future [9,13,14,16]. A randomized controlled trial could show that an ePROs-based telehealth follow-up strategy can achieve similar disease control as conventional follow-up [17]. This need-adapted follow-up strategy could set precious resources free, which could thus reduce the diagnostic and monitoring delay in RMD patients. A parallel collection of ePROs may facilitate the integration of ePROs into the daily routine. Importantly, wearable-based ePROs produce continuous data sets and can accurately predict disease flares in patients with rheumatoid arthritis and axial spondyloarthritis [18].

Digital registries are crucial for RMD research. Only in a collaborative effort [19] significant amounts of data can be gathered to improve knowledge among rheumatologists. Registries are urgently needed to support RMD patients’ management based on evidence. Registries have helped to provide knowledge for rare diseases, such as myositis [20], specific patient scenarios, such as pregnancy [21], and the safety of biologic therapy [22]. Unfortunately, the data collection approach of most patient registries relies on cumbersome active and redundant manual data entry, which frequently lacks precision or completeness [23]. Values can be inconsistent, out of range, or even completely made up. Most importantly, data entry and employment often stop with the end of financial support [24]. Fortunately, most RMD patients are willing to share their data for research purposes [14].

## RheumaDatenRhePort Patient Pathway

**Figure 1:**
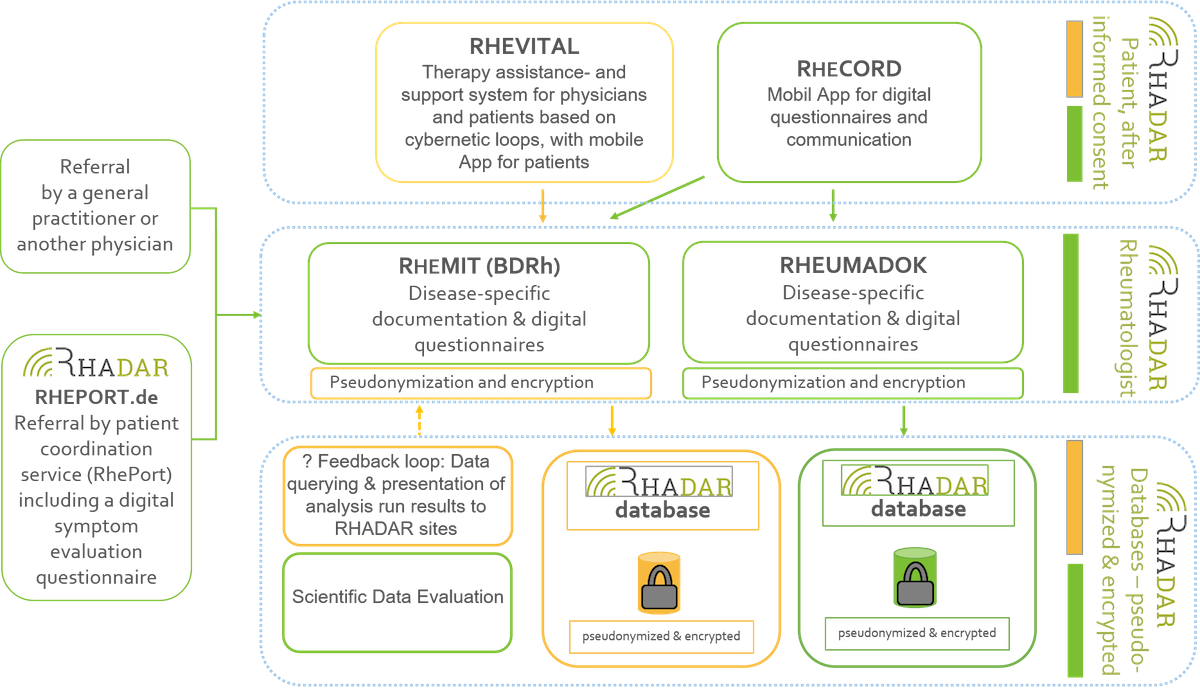
RHADAR patient and data pathway. Green boxes resemble established parts of the RHADAR network. Orange boxes are in preparation. RHADAR: RheumaDatenRhePort.

The RheumaDatenRhePort (RHADAR) registry (Figure 1) is based on a framework of complementary software modules covering the patient pathway from symptom onset through the disease course.

### RhePort: Digital Gate to Rheumatology Care

**Figure 2:**
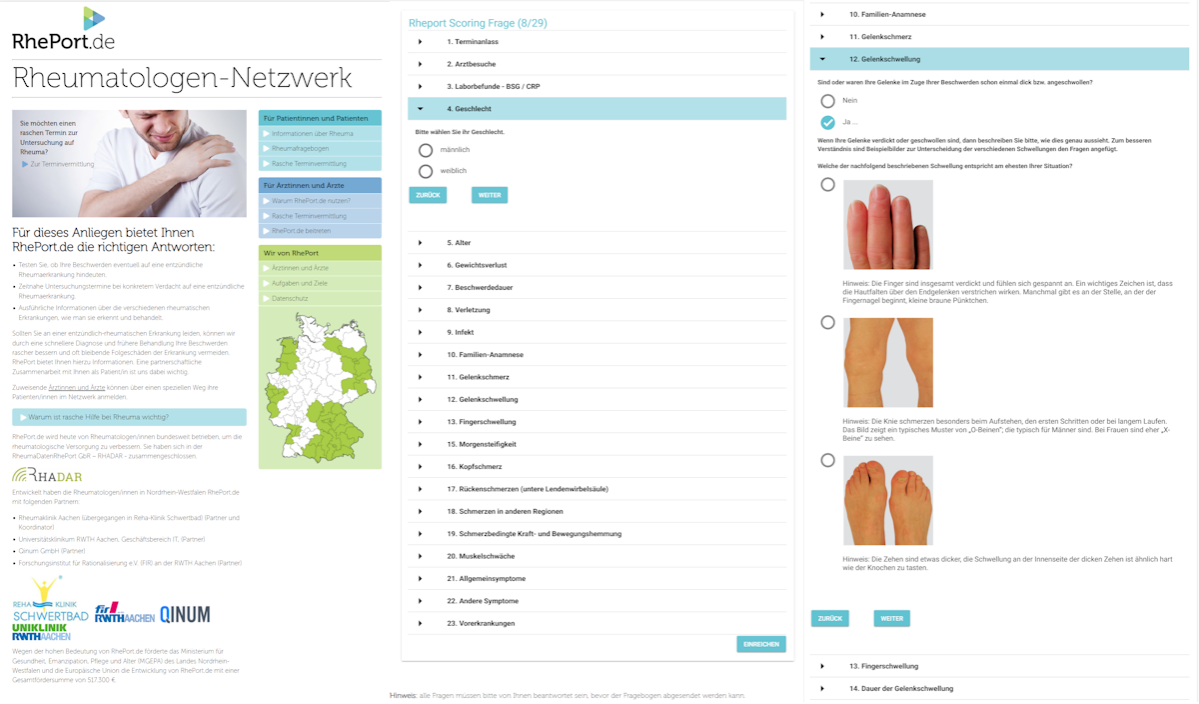
Screenshots of RhePort.

RHADAR provides low burden direct digital access to a growing network of participating rheumatology sites. The gate module toward RHADAR rheumatology care is RhePort (Figure 2) [25]. Online registration is necessary to use RhePort, and users need to actively consent to the addition of their data to the RHADAR registry.

RhePort currently consists of a questionnaire that takes on average 9 minutes to complete [26]. It includes necessary demographic information, current symptoms, and already collected laboratory reports. Based on the patient’s answers, RhePort calculates the RMD risk. The implemented algorithm refers to a weighted sum score. Individual weights were derived by consensus and the work experience of the RhePort founders. Based on the RhePort RMD risk score, an individual triage level is determined. Currently, there are four triage levels as follows: (1) very urgent, (2) urgent, (3) intermediate, and (4) unlikely. Based on the corresponding level, the patient gets access to locally available RHADAR rheumatology site appointments.

The questionnaire threshold focuses on sensitivity, that is, omitting false-negative ratings. Furthermore, participating rheumatology sites are encouraged to use RhePort only on a complimentary basis. Proft et al recently showed that an online self-referral strategy can successfully be used in addition to the traditional physician-based referral [27]. By including patients based on symptoms, at-risk RMD patients are included in the registry as well. In an interim analysis of a head-to-head randomized controlled trial, the diagnostic accuracy of RhePort was similar to an artificial intelligence–based symptom checker regarding the presence of RMDs [26].

### RheCORD: Digital Companion App

**Figure 3:**
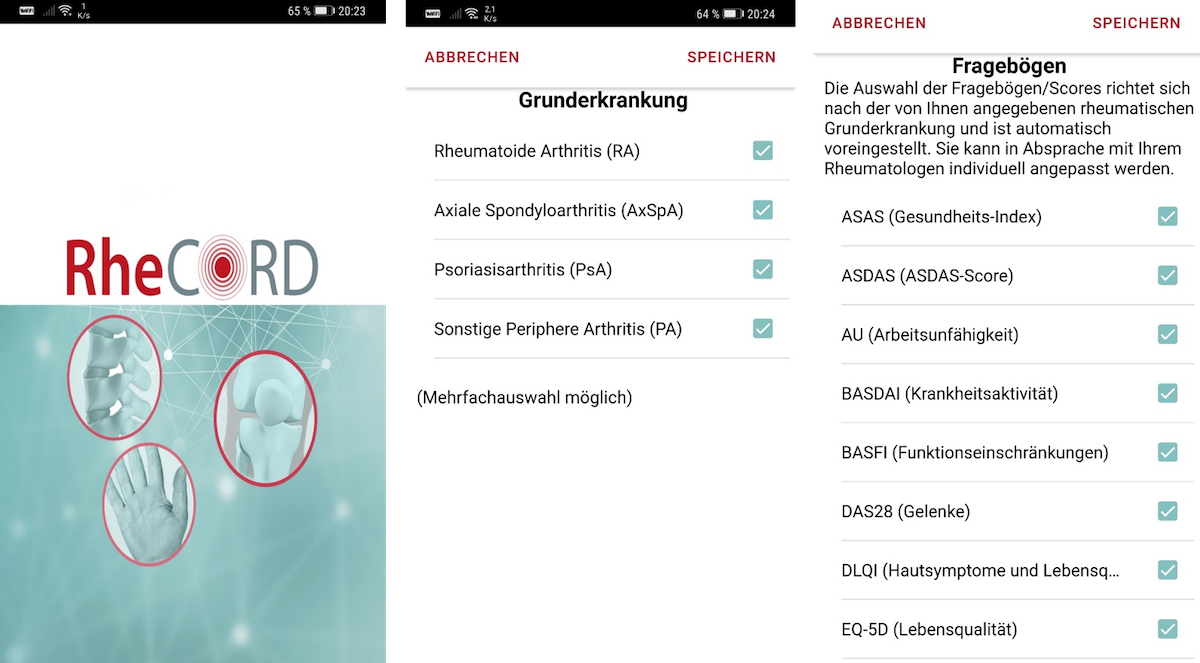
Screenshots of RheCORD.

RheCORD (Figure 3) [28] is a rheumatological medical app currently being developed to enable the collection of ePROs. RheCORD gives insights into the patient’s symptom state between consultations or before an upcoming appointment on the same day. The ePROs to be collected can individually be determined by the patient [14,29] and local rheumatology site [13] via shared decision-making. The app provides multiple optional secondary features, such as medication and appointment reminders. Feature selection and app development are carried out with respect to guidelines [30,31], app analysis [31], and patient insights [14,29]. RheCORD is based on the previous CE-certified medical apps RheumaLive, AxSpALive, and PsALive. RHADAR sites provide a free soft token and password to the patient via a QR code to enable encrypted data transfer to the site.

### RheumaDok

**Figure 4:**
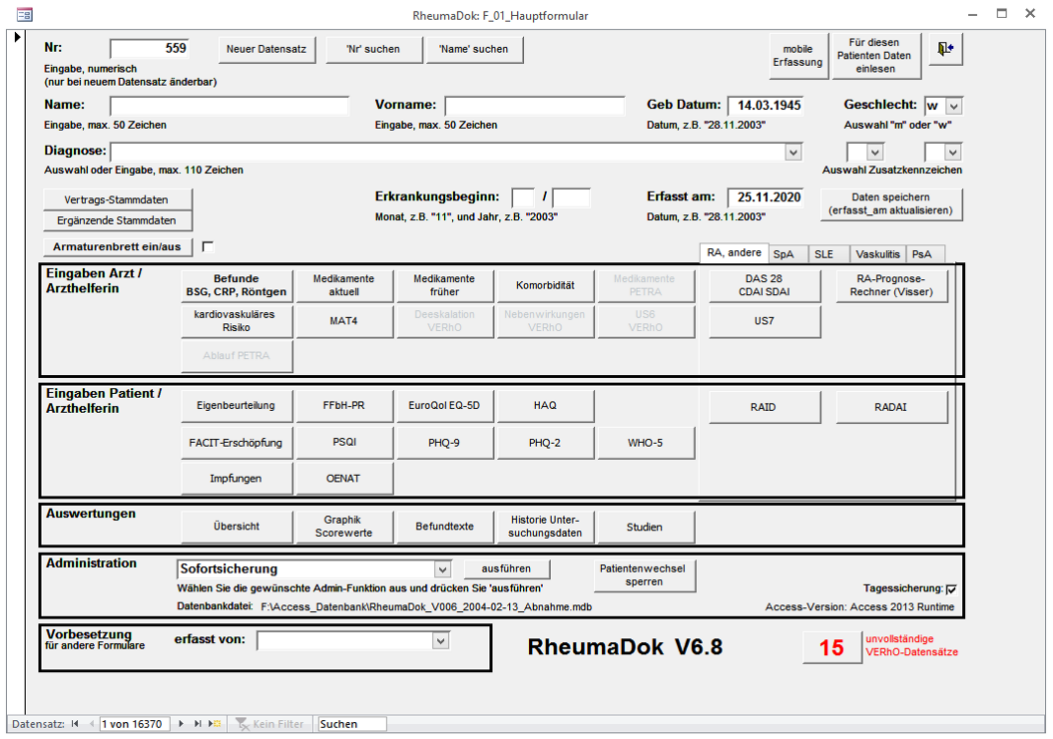
Screenshot of RheumaDok.

RheumaDok (Figure 4) is the current core application used by the RHADAR system for patient data collection. It is a Microsoft Access (Microsoft Corp) database developed in Visual Basic for Applications and is available free of charge for rheumatologists in Germany who are members of the German Rheumatologists’ Professional Association (Berufsverband Deutscher Rheumatologen [BDRh]). This database was developed by the Nils Körber und Joachim Elgas GbR. In the months ahead, RheumaDok will be replaced by RheMIT.

### RheMIT: Clinical Documentation Software

**Figure 5:**
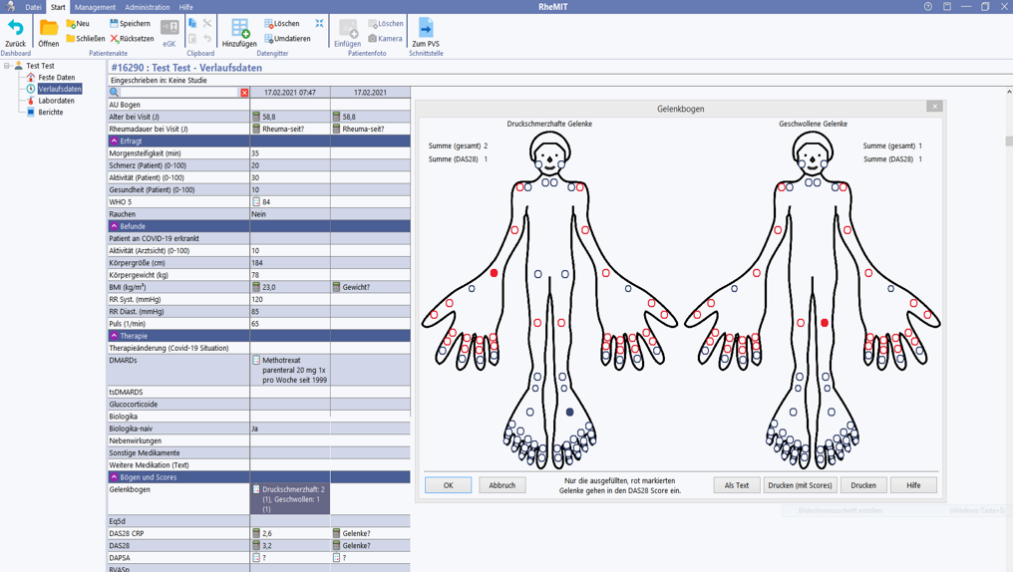
Screenshot of RheMIT.

RheMIT (Figure 5) is currently becoming the standard clinical documentation software for rheumatologists in Germany. The software has been developed and adapted by itc-ms.de for the German Rheumatologists’ Professional Association (BDRh) [7]. The software offers standardized entry masks to document the patient’s disease course at each consultation, including standard disease activity measures, general or disease-specific PROs, demographic characteristics, comorbidities, and current as well as previous antirheumatic treatments. If patients provide their consent, pseudonymized information will be uploaded to the RHADAR registry.

### RHADAR Registry

All information from RheCORD and local RheumaDok database instances are pseudonymized at each participating site. Subsequently, data are merged in a mutual database. The responsible data protection authority approved the current RHADAR database environment to ensure data privacy according to legislation. Patients participating in the RHADAR network have to complete an informed consent form before their initial data entry. Participation is voluntary for each patient. Participants and patients refusing to participate are treated equally, following national guidelines and rheumatologic care standards in Germany. As of September 2020, 22 rheumatologists at eight sites provided data to the RHADAR registry, including a total of 15,908 patients.

## RHADAR Outlook

RHADAR software modules are continuously being improved based on new technological standards, user preferences, and scientific literature. For instance, a machine-learning approach to improve the triage performance of the RhePort algorithm is being evaluated based on current data. The implementation of an objective, standardized, and evidence-based triage algorithm seems crucial. Additionally, a complementary software module, RheVITAL [32], is under development in cooperation with the largest German patient organization, the German Rheumatism League (“Deutsche Rheuma-Liga”), to facilitate RMD disease management. Several registry analyses are being carried out and are set to directly impact future RMD management. A new database is currently under construction, and it will include data from all current and future software modules. Growing awareness of the integrative RHADAR system and its distribution will foster networking of rheumatology sites.

## Abbreviations

BDRh: Berufsverband Deutscher Rheumatologen (German Rheumatologists’ Professional Association)
ePROs: electronic patient-reported outcomes
RHADAR: RheumaDatenRhePort
RMD: rheumatic and musculoskeletal disease
